# The Treatment of Scarlet Fever by Antiseptic Inunction

**Published:** 1893-07-01

**Authors:** Bertram Rogers

**Affiliations:** Clifton


### Editor's Letter-Box.

[Our correspondents are reminded that prolixity is a great bar to publication, and that brevity of style and conciseness of statement greatly facilitate early insertion.]

### THE TREATMENT OF SCARLET FEVER BY ANTISEPTIC INUNCTION.

Sir,—I quite agree with Mr. Curgenven that my cases are too few to condemn the use of his particular disinfectant, but I may be allowed to point out that failure to obtain certain results with a drug is often of more value than success.

Mr. Curgenven's paper in the *Medical Magazine* is entitled "The Treatment and Disinfection of Scarlet Fever by Antiseptic Inunction," and on page 739 he states that he has had no instance of infection among the cases treated by this method, *i.e.*, inunction. I therefore concluded, with I think you will allow justice, that though Mr. Curgenven "advises" the internal administration of this drug, inunction alone is not sufficient to prevent the spread of the disease among susceptible people. I fail to follow Mr. Curgenven's argument that, as I was unable to discover the source of infection, my treatment must almost of necessity result in failure. That it did result in failure I admit, but so would most cases if the practitioner being unable to discover the source of infection is an element leading to failure. The servant without doubt caught the disease from one of the patients, and yet she had been for 21 days constantly in an atmosphere charged with eucalyptus, for the inunction was carried on twice daily for over six weeks. Can there, I ask, be any disinfecting power in such a body as this oleusaban, when patients exposed to its vapour for three weeks yet take the disease which the disinfectant is said to specially protect from?

That no one else in the house fell a victim to the disease was due to the prompt isolation of the first case. I know of a medical man who puts his cases of scarlet fever on the upper floors because, he says, the infective particles or germs are lighter than air, and so ascend. His treatment is very successful, and the infection does not spread to the lower regions of the house.

In one of the two passages which Mr. Curgenven finds a difficulty to reconcile, I might have made my meaning more clear by adding after "fever" the words "and exposed frequently to the infection," but I do not think the majority of your readers will find it much trouble to arrive at my meaning without this addition.

The oleusaban supplied to me was certainly not "as clear as spirit." It was as white as milk.—I am, yours truly,

Clifton, June 20th. Bertram Rogers.

